# Development and validation of a predictive model for postoperative urinary retention following pelvic organ prolapse surgery: a retrospective study

**DOI:** 10.3389/fmed.2026.1851119

**Published:** 2026-06-24

**Authors:** Juan Zhang, Jie Cui, Fei Li, Yanling Li, Yana Gao, Gaijing Wang

**Affiliations:** 1 School of Nursing, Hebei University, Baoding, China; 2 Department of Obstetrics, Affiliated Hospital of Hebei University, Baoding, China; 3 Department of Nursing, Affiliated Hospital of Hebei University, Baoding, China; 4 Department of Obstetrics and Gynecology, Affiliated Hospital of Hebei University, Baoding, China; 5 Department of Gynecology, Affiliated Hospital of Hebei University, Baoding, China

**Keywords:** Charlson Comorbidity Index, machine learning, predictive model, prolapse surgery, urinary retention

## Abstract

**Objective:**

Postoperative urinary retention (POUR) is a common complication following pelvic organ prolapse (POP) repair surgery, significantly impacting patient recovery. Establishing an effective predictive model facilitates individualized risk assessment and postoperative management.

**Methods:**

This retrospective study included 698 patients who underwent POP surgery. The dataset was chronologically divided: patients from January 2020 to June 2024 formed the training group (*n = 505*), and those from July 2024 to December 2025 formed the temporal external validation group (*n = 193*). Variables were determined using univariate analysis and least absolute shrinkage and selection operator (LASSO) regression, and six machine learning models were developed and compared.

**Results:**

Among 698 patients, 176 (25.21%) developed POUR. Following feature screening, 10 predictors were included in the models: degree of anterior pelvic prolapse (point Ba), bladder neck mobility, Charlson Comorbidity Index (CCI), preoperative venous thromboembolism (VTE) risk score, preoperative blood glucose (GLU), postoperative analgesia, Parity, and three types of surgical procedures. In the temporal validation cohort, the gradient boosting decision tree (GBDT) model achieved an AUC of 0.848 and showed a modest but statistically significant improvement over logistic regression (ΔAUC = 0.0397; 95% CI: 0.0165–0.0629; Holm-adjusted *P* = 0.0032), with stable bootstrap performance and higher net benefit across thresholds of 0.10–0.50.

**Conclusion:**

These findings suggest that the GBDT model has potential for POUR prediction in this single-center setting, but multicenter prospective validation is required before clinical implementation.

## 1 Introduction

Pelvic organ prolapse (POP) is a common benign condition in women that can cause symptoms such as vaginal pressure, urinary and bowel dysfunction, significantly impacting patients’ quality of life. The prevalence of POP based on physical examination ranges from 41 to 50%, although symptomatic POP is less common (3–6%) (1). Common risk factors for POP include advancing age, high parity (childbirth), and increased intra-abdominal pressure (2). For symptomatic patients with POP, the primary treatment is surgical intervention to restore the normal anatomical structure of the pelvic floor. POUR is a common complication following POP repair surgery, with an incidence ranging from 2.5 to 45% (3–5). If not promptly identified and managed, it may lead to complications such as urinary tract infections, impaired bladder function, and failed surgical repair. Moreover, the occurrence of POUR may prolong the length of hospital stay, thereby severely impairing postoperative recovery and quality of life (5–7).

Although POP and POUR share some risk factors (e.g., age and parity), their risk profiles are distinctly different. POUR is influenced by a combination of factors, including diabetes, surgical procedure types, intraoperative fluid administration, and operative duration, rather than by the anatomical severity of prolapse alone. Therefore, predicting POUR requires an independent model. Several common risk factors for POUR following gynecological surgery have been identified in previous studies. Beyond the factors mentioned above, These include advanced age, more severe prolapse stages, preoperative urinary dynamic abnormalities, Pelvic Organ Prolapse Quantification (POP-Q) classification, and surgical variables (8–11). Operative-related factors are particularly critical, such as high intraoperative blood loss, prolonged surgical duration, and certain procedures (e.g., anterior repair, anti-urinary incontinence surgery, and hysterectomy) are strongly associated with an increased risk of POUR (12, 13). Anglim et al. developed a POUR risk calculator for vaginal pelvic floor surgery that incorporates variables such as age, prolapse grade, urinary flow parameters, and surgical method, achieving a C-index of 0.73 (14). However, this model was developed using conventional logistic regression and did not incorporate systematic comorbidity assessment. To address this gap, our study introduces the Charlson Comorbidity Index (CCI) as a standardized measure of overall comorbidity burden and, more importantly, systematically evaluates six machine learning approaches ranging from generalized linear models (e.g., logistic regression) to tree-based ensemble methods to more flexibly capture potential nonlinear relationships between the predictors and the outcome. The Charlson Comorbidity Index (CCI) is a valuable tool for evaluating the severity of comorbidities and long-term prognosis (15), and it has been established as an independent predictor of complications following various surgical procedures (16). Nevertheless, its predictive value for POUR in pelvic floor surgery has yet to be thoroughly validated.

The study aims to enhance the understanding of CCI’s function in predicting POUR by integrating it with surgical factors, demographic characteristics, and preoperative clinical signs to develop a comprehensive risk prediction model. Through systematic retrospective analysis and model validation, we aim to develop a predictive tool with favorable clinical applicability, providing a reference for preoperative risk assessment and individualized postoperative management.

## 2 Materials and methods

### 2.1 Study design and participants

This study is a retrospective analysis of the electronic medical records from a tertiary-level Class A hospital in Hebei Province. It aims to develop and validate a machine learning model to predict the risk of POUR following POP surgery. The study’s implementation and reporting strictly adhere to the Transparent Reporting of Individualized Prognostic or Diagnostic Multivariate Prediction Models (TRIPOD + AI) guidelines (17), ensuring that the methods, results, and conclusions are comprehensive, accurate, and transparent. Inclusion criteria: (1) Symptomatic POP as determined by a quantitative POP scoring system; (2) Age ≥ 18 years; (3) Undergoing transvaginal pelvic floor repair surgery; (4) Availability of comprehensive clinical medical documentation. Exclusion criteria: (1) Persistent voiding dysfunction, neurogenic bladder, or preexisting urinary retention; (2) Requirement for long-term indwelling urinary catheterization before surgery; (3) History of significant pelvic floor procedures that could affect urinary function, such as previous anti-urinary incontinence surgery; (4) Missing important predictor variables or outcome data in more than 20% of medical records; (5) Preoperative use of medications that could significantly affect bladder function. The authors are accountable for all aspects of the work in ensuring that questions related to the accuracy or integrity of any part of the work are appropriately investigated and resolved. This study was conducted in accordance with the Declaration of Helsinki and was approved by the Ethics Committee of the Affiliated Hospital of Hebei University (Approval No.: HDFY-LL-2021-235). Because of the retrospective nature of the research, the requirement for informed consent was waived.

### 2.2 Data collection

This study collected medical records from 734 patients who underwent transvaginal repair surgery for symptomatic POP at Hebei University Affiliated Hospital between 2020 and 2025. POP was diagnosed and staged using the POP-Q system. All examiners were attending gynecologists who had received standardized training and followed a consistent institutional measurement protocol. Based on a literature review (18–20), preoperative clinical history, laboratory indicators, and sociodemographic variables were extracted from electronic medical records through retrospective review. including age, body mass index (BMI), parity, age at first birth (AFB), CCI, POP-Q, past medical history, and history of previous surgeries and surgical procedures. BMI was calculated by dividing an individual’s weight (in kilograms) by the square of their height (in meters). The CCI was determined using the patient’s age and number of comorbidities. Gynecologists at our institution employed internationally standardized methods to measure POP-Q points (Aa, Ba, and C) prior to surgery. During the Valsalva maneuver, a disposable measuring ruler was used to record the position of each point (in centimeters) relative to the hymenal margin. All data for this investigation were obtained from the hospital’s medical record system. Medical records with more than 20% missing data were excluded from the analysis, a total of 698 patients were finally enrolled in this study. Of the 734 patients initially identified, 36 were excluded: 10 received conservative treatment, 12 had preoperatively diagnosed voiding dysfunction, and 14 had > 20% missing predictor values (per-sample exclusion). The final analytic cohort comprised 698 patients. In this cohort, the proportion of missing data for each individual variable ranged from 0 to 3.3% (Supplementary Table S1). Missing values for the remaining variables were processed as follows: multiple imputation was used for continuous variables, while the mode was adopted to impute missing values for categorical variables. Specifically, multiple imputation was performed using the MICE algorithm with 20 iterations, and results were pooled according to Rubin’s rules. Missingness was assumed to be missing at random (MAR), as it could be predicted by observed baseline characteristics.

The clinical diagnosis of POUR often requires a comprehensive evaluation that combines objective data with the patient’s subjective symptoms. In practice, assessment thresholds and procedures vary. Currently, the primary assessment methods are the reverse flow test or spontaneous voiding test, supported by ultrasound or catheter measurement of post-void residual urine volume (21–23). Standardized operational definitions were applied to ensure the validity of the study’s conclusions. POUR was defined as a post-void residual volume > 150 mL measured by bladder scan after initial catheter removal, consistent with widely accepted clinical guidelines (24). This threshold was used as the uniform diagnostic criterion throughout the study period. This unified standard seeks to reduce biases caused by variations in clinical practice, thereby ensuring the impartiality and comparability of the research findings.

### 2.3 Data encoding

This study consistently coded and preprocessed all predictor variables. Age, BMI, number of pregnancies, number of births, AFB, preoperative VTE score, preoperative blood glucose (GLU), CCI, and POP-Q were treated as continuous variables. The POP-Q includes measurements at points Aa, Ba, and C, with all variables incorporated as raw values. All categorical variables were coded as dichotomous variables (0 = no, 1 = yes), including diabetes, hypertension, coronary heart disease, history of cesarean section, history of pelvic surgery, bladder neck mobility (0 = normal, 1 = increased), postoperative analgesia method (0 = none, 1 = non-opioid analgesics), and POUR. The type of surgery was coded as multiple binary indicators, including hysterectomy, anterior wall repair, posterior wall repair, sacrospinous ligament suspension, and Manchester procedure (all coded as 0 = not performed, 1 = performed).

### 2.4 Statistical analysis

**FIGURE 1:**
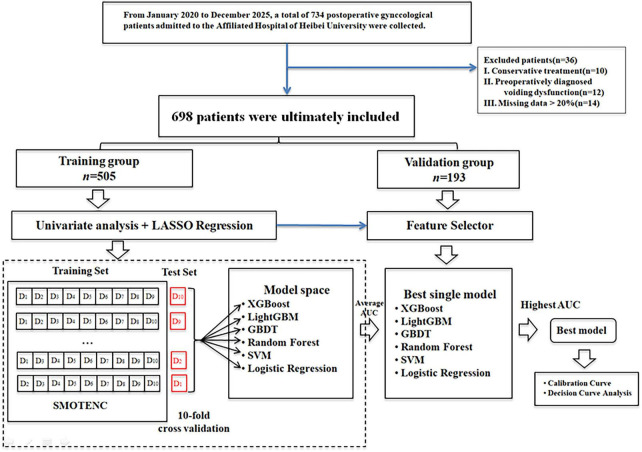
Overall flow diagram.

Statistical analysis was performed using Python version 3.13.11. Descriptive analyses were conducted on the clinical baseline characteristics of the enrolled patients. To assess model generalizability, the dataset was chronologically divided based on admission date. Patients admitted between January 2020 and June 2024 were assigned to the training group (*n* = 505), and those admitted between July 2024 and December 2025 served as the temporal external validation group (hereinafter, the validation group) (*n* = 193). In the training group, we first conducted univariate analyses: for categorical variables, Fisher’s exact test or the chi-square test were used; for continuous variables, Student’s *t*-test was employed. variables significantly associated with the outcome were screened out, as shown in Table 1. To further reduce dimensionality, feature selection was performed using least absolute shrinkage and selection operator (LASSO) regression with 10-fold cross-validation, yielding a shrunk estimator, Only 10 variables with non-zero coefficients were retained. Based on the filtered features, we compared six predictive models: eXtreme gradient boosting (XGBoost), light gradient boosting machine (LightGBM), gradient boosting decision tree (GBDT), Random Forest, Logistic Regression, and Support Vector Machine (SVM). The optimal hyperparameters for each model were determined using the Bayesian optimization algorithm, with the evaluation metric being the mean area under the curve (AUC) value from 10-fold cross-validation on the training group. Given that positive samples are fewer than negative samples, during cross-validation, SMOTENC algorithm was applied to perform oversampling on the positive samples in the 9-fold data used for model training in each iteration, until the ratio of positive samples to negative samples reached 1:2. To evaluate the robustness of the final GBDT model to the oversampling strategy, an additional sensitivity analysis was conducted (Supplementary Table S2). Finally, the optimal model was selected from multiple candidates based on the AUC of the receiver operating characteristic (ROC) curve on the Validation Group. The calculation of CI differs between the training group and the validation group. For the training group, 10-fold cross-validation was used to evaluate the stability of each model, and the 95% CI of the mean AUC was calculated using the corrected *t*-test proposed by Grandvalet and Bengio (25), which accounts for the dependence among the validation folds and yields a more conservative interval estimate with controlled type-I error. For the validation group, the bootstrap method with 1,000 resamples was employed to calculate the 95% CI of the AUC, thereby quantifying the sampling uncertainty. The optimal model was then comprehensively evaluated using calibration curves and decision curves (DCA), with the overall process is illustrated in Figure 1. To support model selection, additional comparative analyses were performed in the temporal validation cohort. Pairwise AUC comparisons between GBDT and the other five models were conducted using DeLong tests with Holm adjustment. To evaluate the stability of GBDT versus logistic regression, 1,000 bootstrap resamples were generated to estimate differences in AUC and Brier score, with 95% confidence intervals reported. Threshold sensitivity and clinical utility were assessed via decision curve analysis, comparing net benefit differences (GBDT minus logistic regression) at thresholds of 0.10, 0.20, 0.30, 0.40, and 0.50, with 95% CIs estimated from bootstrap resamples.

**TABLE 1:** Baseline table (training set grouped by occurrence of urinary retention).

Variable	Level	Overall *N* = 505	Postoperative urinary retention		*p-*value	Holm-adjusted *p*-value
			No (*N* = 378)	Yes (*N* = 127)		
Postoperative analgesia	0	142 (28.1%)	121 (32.0%)	21 (16.5%)	0.001	0.013
	1	363 (71.9%)	257 (68.0%)	106 (83.5%)		
Bladder neck mobility	0	175 (34.7%)	159 (42.1%)	16 (12.6%)	< 0.001	<0.001
	1	330 (65.3%)	219 (57.9%)	111 (87.4%)		
Cesarean	0	500 (99.0%)	373 (98.7%)	127 (100.0%)	0.337	1.000
	1	5 (1.0%)	5 (1.3%)	0 (0.0%)		
Diabetes	0	426 (84.4%)	339 (89.7%)	87 (68.5%)	< 0.001	<0.001
	1	79 (15.6%)	39 (10.3%)	40 (31.5%)		
Hypertension	0	227 (45.0%)	183 (48.4%)	44 (34.6%)	0.009	0.085
	1	278 (55.0%)	195 (51.6%)	83 (65.4%)		
Coronary heart disease	0	478 (94.7%)	362 (95.8%)	116 (91.3%)	0.091	0.635
	1	27 (5.3%)	16 (4.2%)	11 (8.7%)		
Surgical procedures						
Anterior colporrhaphy	0	249 (49.3%)	220 (58.2%)	29 (22.8%)	< 0.001	<0.001
	1	256 (50.7%)	158 (41.8%)	98 (77.2%)		
Posterior colporrhaphy	0	188 (37.2%)	169 (44.7%)	19 (15.0%)	< 0.001	<0.001
	1	317 (62.8%)	209 (55.3%)	108 (85.0%)		
Hysterectomy	0	390 (77.2%)	275 (72.8%)	115 (90.6%)	< 0.001	<0.001
	1	115 (22.8%)	103 (27.2%)	12 (9.4%)		
Manchester	0	439 (86.9%)	314 (83.1%)	125 (98.4%)	< 0.001	<0.001
	1	66 (13.1%)	64 (16.9%)	2 (1.6%)		
SSLF	0	341 (67.5%)	274 (72.5%)	67 (52.8%)	< 0.001	<0.001
	1	164 (32.5%)	104 (27.5%)	60 (47.2%)		
Pelvic operation	0	298 (59.0%)	219 (57.9%)	79 (62.2%)	0.458	1.000
	1	207 (41.0%)	159 (42.1%)	48 (37.8%)		
**Stage**						
2		7 (1.4%)	6 (1.6%)	1 (0.8%)	0.171	1.000
3		418 (82.8%)	306 (81.0%)	112 (88.2%)		
4		80 (15.8%)	66 (17.5%)	14 (11.0%)		
CCI		4.00 (3.00–5.00)	4.00 (3.00–5.00)	5.00 (4.00–5.00)	< 0.001	<0.001
Age		5.30 (4.90–5.90)	5.30 (4.90–5.70)	5.30 (5.10–6.40)	0.005	0.049
GLU		5.30 (4.90–5.90)	5.30 (4.90–5.70)	5.30 (5.10–6.40)	0.005	0.049
BMI		25.20 (23.70–27.20)	25.20 (23.80–27.20)	25.20 (23.50–27.25)	0.800	1.000
Gravida		3.00 (2.00–4.00)	3.00 (2.00–4.00)	3.00 (2.00–4.00)	0.053	0.428
Parity		2.00 (2.00–3.00)	2.00 (2.00–3.00)	2.00 (2.00–3.00)	< 0.001	0.009
AFB		24.00 (23.00–25.00)	24.00 (23.00–25.00)	24.00 (23.00–26.00)	0.309	1.000
**POP-Q**						
Aa		1.00 (0.00–2.00)	1.00 (0.00–2.00)	1.00 (0.00–2.00)	0.115	0.692
Ba		3.00 (1.50–4.00)	3.00 (1.00–4.00)	3.50 (2.50–4.00)	< 0.001	<0.001
C		2.00 (−0.50–4.00)	2.00 (−0.50–4.00)	0.50 (−0.50–4.00)	0.194	0.971
Preoperative VTE		5.00 (4.00–5.00)	5.00 (4.00–5.00)	5.00 (5.00–6.00)	< 0.001	<0.001

Continuous variables are presented as median (interquartile range) and were compared using the Mann-Whitney U test because they did not satisfy the normality assumption. Categorical variables are presented as n (%) and were compared using the chi-square test or Fisher’s exact test, as appropriate. *P*-values were adjusted for multiple comparisons using the Holm method. VTE, venous thromboembolism; CCI, Charlson Comorbidity Index; POP-Q, Pelvic Organ Prolapse Quantification; SSLF, sacrospinous ligament suspension.

## 3 Results

### 3.1 Patient characteristics

This retrospective study included 698 patients who underwent POP surgery, all of whom were valid cases. Within the training group used for model development, 378 patients (74.9%) did not experience POUR, while 127 patients (25.1%) did experience POUR. Patients were grouped based on the occurrence of urinary retention, and baseline characteristics compared as shown in Table 1. Baseline characteristics were compared between patients with and without POUR, postoperative analgesia, bladder neck mobility, diabetes, surgical approach, age, GLU, parity, Ba, CCI and preoperative VTE. These 14 variables were all statistically significantly associated with the occurrence of POUR (*P* < 0.05).

In terms of demographic characteristics and preoperative status, the mean age of patients in the POUR group was significantly higher than that in the non-retention group, and the mean number of previous deliveries was greater in the retention group. Patients in the urinary retention group had a higher mean number of previous deliveries. The urinary retention group exhibited significantly higher prevalence rates of diabetes and hypertension, along with higher mean preoperative fasting blood glucose levels. Among patients undergoing anterior pelvic reconstruction, posterior pelvic reconstruction, and sacrospinous ligament fixation, the incidence of urinary retention was significantly higher. While those receiving postoperative analgesia had a significantly higher incidence of urinary retention. Based on POP assessment, restricted bladder neck mobility was significantly associated with an increased risk of POUR. The urinary retention group exhibited significantly larger mean values for the Aa and Ba points in the preoperative POP-Q staging, indicating more severe prolapse. Regarding other indicators, the preoperative venous thromboembolism risk score and CCI were both significantly higher in the urinary retention group.

### 3.2 Feature selection

**FIGURE 2:**
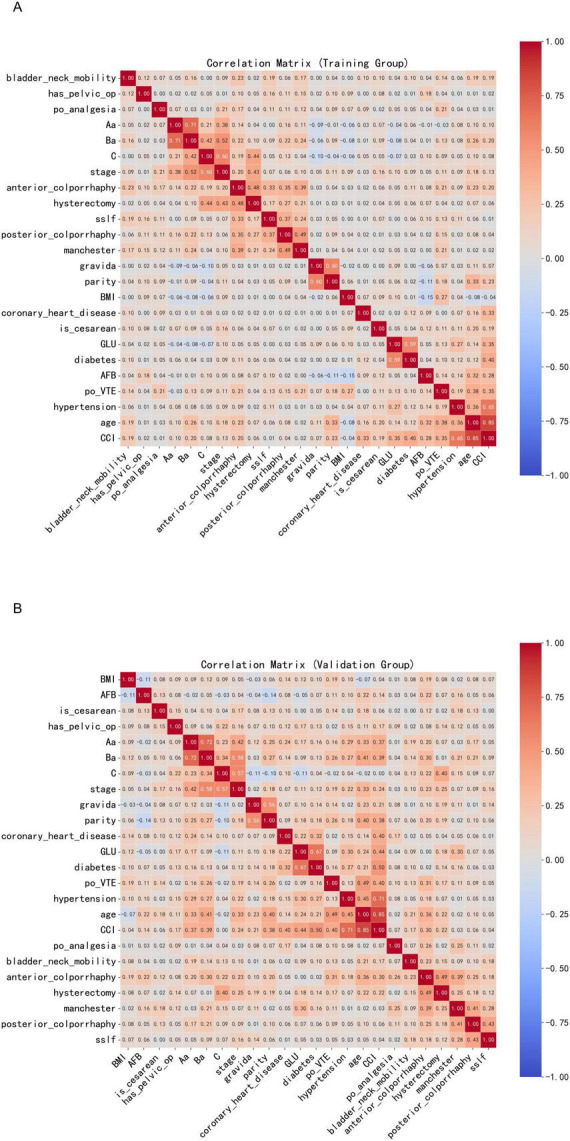
Feature correlation matrix for **(A)** the training group and **(B)** temporal validation group.

Based on the initial screening through univariate analysis, to construct a more streamlined and robust predictive model, correlation analysis was performed on all variables, as shown in Figure 2. Pearson’s correlation coefficient was used to measure associations between continuous variables. Cramér’s V, based on the chi-square test, was applied to categorical variables. The correlation ratio (η^2^, Eta squared) was employed to assess relationships between continuous and categorical variables. It was observed that Aa and Ba exhibited a high correlation; therefore, the relatively insignificant Aa was removed. During CCI calculation, a linear combination of Age and Diabetes is employed, incorporating information from both variables. CCI demonstrates a strong correlation with Age; thus, Age is removed to retain the more informative CCI. GLU also shows a strong correlation with Diabetes. Since Diabetes is already utilized in CCI calculation, GLU is retained instead. Based on the remaining 13 variables, an embedded feature selection method was employed to reduce the dimensionality of the variables. By constructing a Lasso regression model, L1 regularization penalties were applied to the remaining 13 candidate variables within the training set, thereby automatically achieving variable screening and complexity control. After optimizing the penalty coefficient through 10-fold cross-validation, the model ultimately retained 10 predictor variables with non-zero regression coefficients, which were incorporated into subsequent modeling procedures. This method reduces the risk of model overfitting while ensuring the retention of critical predictive information, thereby enhancing the model’s clinical interpretability and generalization capability.

### 3.3 Model development and validation

Based on the above results, a total of 10 independent risk factors were included: bladder neck mobility, CCI, POP-Q (Ba point), preoperative VTE score, Postoperative analgesia, GLU, parity, and type of surgery (vaginal anterior wall repair, vaginal posterior wall repair, sacrospinous ligament suspension). Input the Training Group containing only these 10 variables into six distinct machine learning models. Employ 10-fold cross-validation and Bayesian optimization algorithms to determine the optimal hyperparameters for each model. Subsequently, evaluate the predictive capabilities of these six models—now tuned to their optimal hyperparameters—using the AUC values on the Validation Group.

**FIGURE 3:**
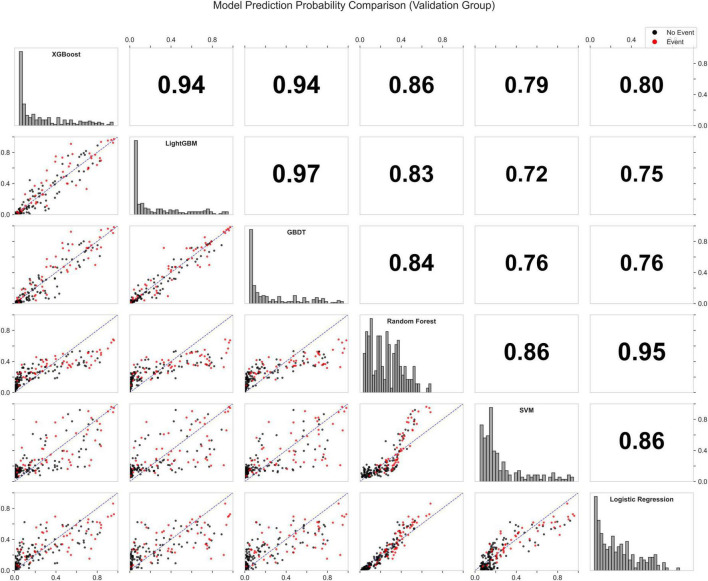
Correlation plot of prediction probability distributions across multiple models on the temporal validation group. This figure illustrates the relationships among POUR prediction probabilities across six distinct models within the development sample. Diagonal: Displays histograms of each model’s predicted probabilities alongside its model name. Lower left (below the diagonal): Presents scatter plots of pairwise model prediction probabilities, with the x-axis representing column model predictions and the y-axis representing row model predictions. Upper right (above the diagonal): Shows Pearson correlation coefficients between each model’s predicted probabilities. Color distinction: Participants with POUR are represented in red, while those without POUR are shown in black. Reference line: The blue diagonal line indicates perfect agreement in predicted probabilities between two models.

**FIGURE 4:**
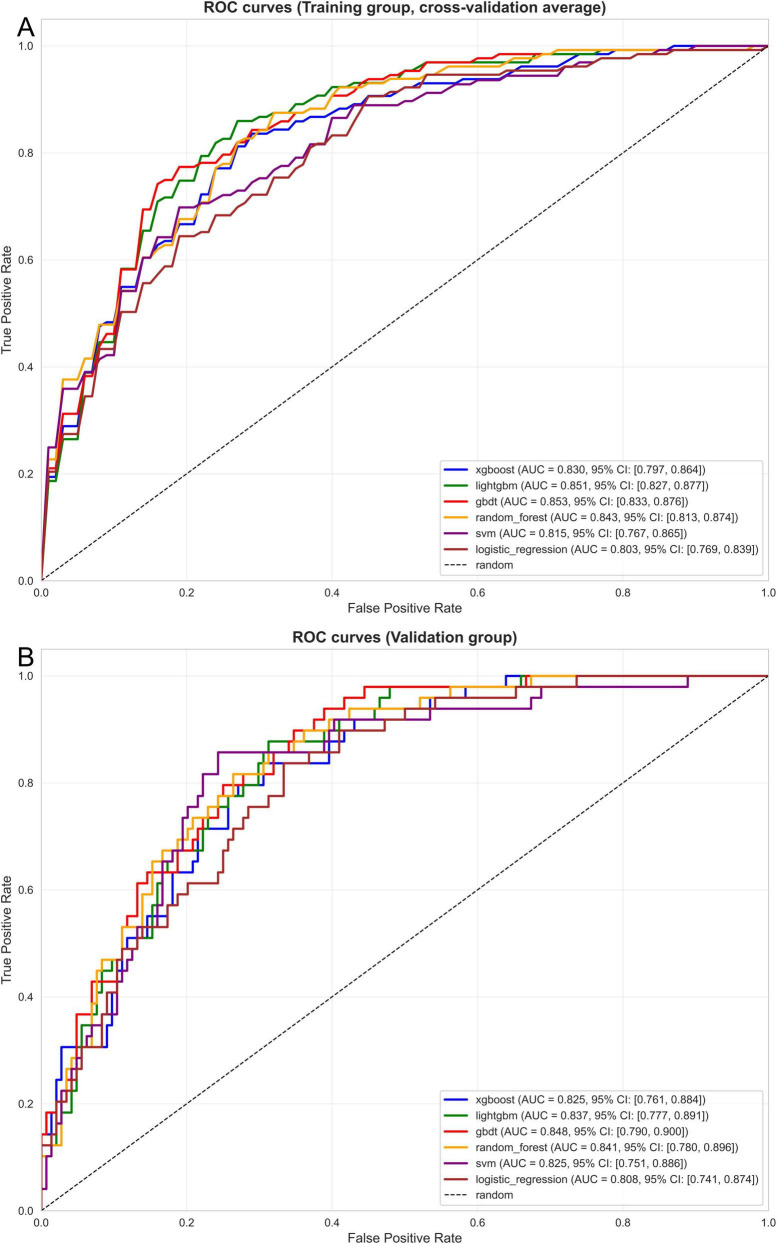
ROC curves and their AUC values for the six models. **(A)** ROC curves in the training group (cross-validation average). **(B)** ROC curves in the temporal validation group.

**FIGURE 5:**
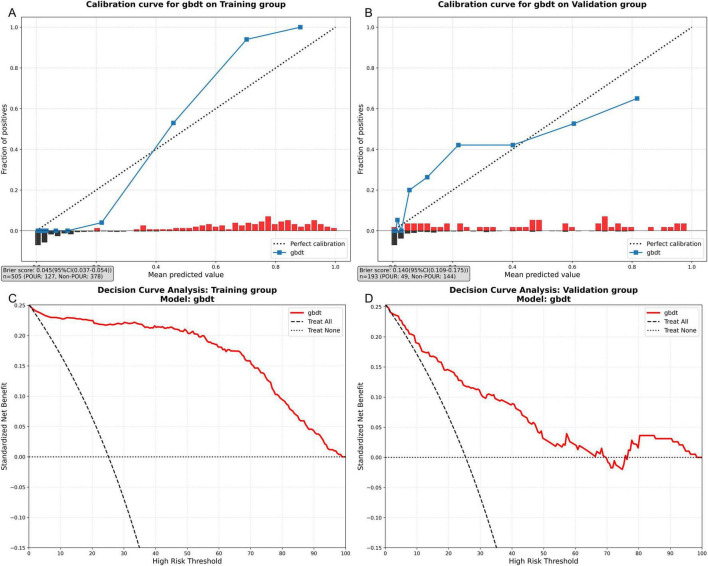
Performance evaluation of the GBDT model. **(A)** Calibration curve in the training group. **(B)** Calibration curve in the temporal validation group. **(C)** Decision curve in the training group. **(D)** Decision curve in the temporal validation group.

The performance of six prediction models was compared, including XGBoost, LightGBM, Random Forest, GBDT, SVM, and Logistic Regression. To assess the correlation among the prediction probabilities of different machine learning models, a correlation analysis of the prediction probabilities from six models on the Validation Group is presented in Figure 3. It can be observed that the predicted probabilities of the four tree-based ensemble models, namely XGBoost, LightGBM, GBDT, and Random Forest, show relatively high correlations. SVM and Logistic Regression, which belong to generalized linear models, also exhibit relatively high correlations. Apart from Random Forest not learning sufficiently well on high probabilities, it is presumed to have poor calibration, other five models were distributed across the entire probability range, with good generalization and calibration. Furthermore, the ROC curves and area under the curve (AUC values) of each model in the Training Group and Validation Group were calculated, as shown in Figure 4, It can be observed that the tree-based ensemble models XGBoost, LightGBM, GBDT, and Random Forest yield higher AUC values on the Validation Group than the generalized linear models SVM and Logistic Regression, among which the GBDT model achieves the highest AUC. The final GBDT model used the following hyperparameters: learning_rate = 0.066, n_estimators = 103, max_depth = 5, min_samples_leaf = 10, min_samples_split = 8, subsample = 0.794. Although the absolute AUC advantage of GBDT over logistic regression was modest (0.848 vs. 0.808), DeLong testing confirmed a significant difference (ΔAUC = 0.0397; 95% CI: 0.0165–0.0629; Holm-adjusted *P* = 0.0032), with GBDT also outperforming XGBoost, LightGBM, SVM, and Random Forest (Supplementary Table S3). Bootstrap resampling (*n* = 1,000) showed stable AUC improvement (bootstrap 95% CI: 0.0204–0.0607) and lower Brier score (difference = −0.0057; 95% CI: −0.0083 to −0.0030) for GBDT (Supplementary Table S4; Supplementary Figure S1). Decision curve analysis further demonstrated higher net benefit for GBDT across thresholds of 0.10–0.50, with bootstrap-estimated differences (Supplementary Figure S2; Supplementary Table S4). To assess the consistency between the predicted risk and the actual observed risk of the GBDT model with the highest AUC value in the Validation Group, a calibration curve was plotted, as shown in Figures 5A,B. The results demonstrate that the predicted probabilities from the GBDT model exhibit high consistency with observed probabilities across the entire risk spectrum. The calibration curve for the Training Group dataset closely aligns with the perfect calibration curve, while in the Validation Group, although the alignment is slightly less precise, it still maintains a high degree of consistency. This indicates the model possesses excellent calibration performance, with highly reliable risk predictions suitable for clinical risk assessment. The clinical net benefit of the assessment model at different risk thresholds was further illustrated by plotting the decision curve analysis (DCA), as shown in Figures 5C,D. It can be seen that within a wide range of risk thresholds on the Validation Group, the net benefit from risk stratification using the GBDT model is significantly higher than that of the Treat All and Treat None strategies. For example, at a risk threshold of 30%, the net benefit of the GBDT model is approximately 0.11, while that of the Treat All strategy is about −0.07. This indicates the model’s ability to effectively identify genuinely high-risk patients, demonstrating strong clinical applicability and decision-support value. Therefore, after comprehensively evaluating the model’s generalization capability, prediction stability, and clinical applicability, GBDT was ultimately selected as the predictive model.

### 3.4 Analysis of optimal model interpretability

**FIGURE 6:**
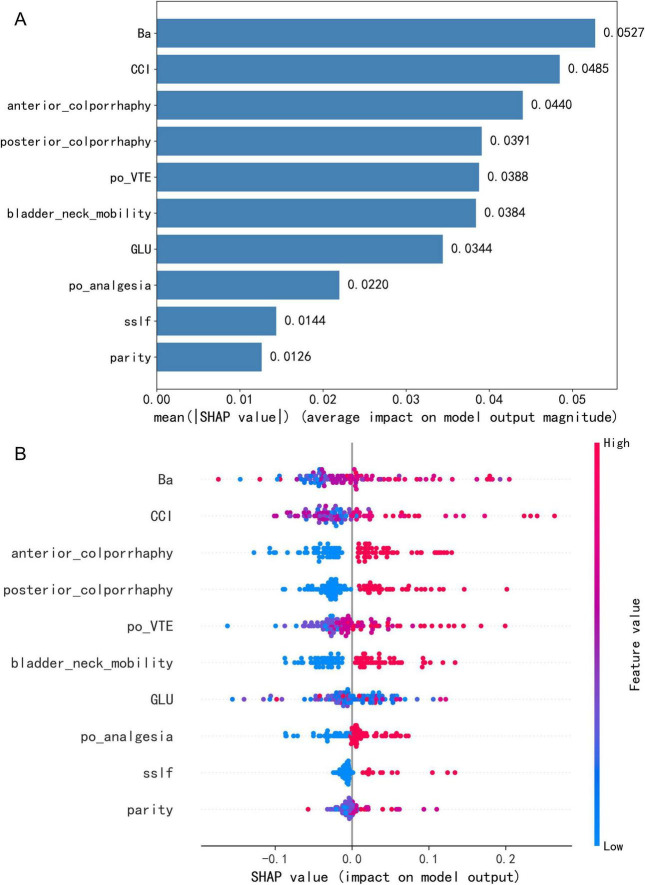
SHAP analysis plots. **(A)** Feature importance. **(B)** Summary plot.

As a method for interpreting machine learning model predictions, SHAP analysis reveals the contribution and direction of each predictive feature toward the risk of POUR (26). As shown in the chart, the feature importance ranking based on average absolute SHAP values (Figure 6A) indicates that the anterior POP degree (Ba point, 0.0527) was the most influential predictor for model output, followed by CCI (0.0485) and anterior colporrhaphy (0.0440). Other important features included posterior colporrhaphy, preoperative VTE score, bladder neck mobility, and others in sequence. The SHAP summary plot further reveals the relationship between feature values and model outputs (Figure 6B). It is noteworthy that all significant features exhibit positive SHAP values in this model, indicating that higher values of these features correlate with an increased risk of urinary retention. The distribution of feature points demonstrates that higher feature values (in red) correspond to more pronounced positive SHAP values, signifying a greater contribution to elevated risk.

### 3.5 Web-based predictive tools

In order to enhance the clinical utility of the predictive model, we have constructed an interpretable web-based clinical prediction tool for predicting POUR risk after POP surgery. The tool can be accessed at: http://111.228.12.177:8501/. Users can input the 10 predictor features (Ba point, CCI, anterior colporrhaphy, posterior colporrhaphy, sacrospinous ligament suspension, preoperative VTE score, preoperative blood glucose, bladder neck mobility, postoperative analgesia, and parity) and obtain an individualized POUR risk probability.

## 4 Discussion

In this study, we retrospectively analyzed the clinical data of 698 patients undergoing POP surgery, and established and validated a risk prediction model for POUR, which included 10 key features: Ba point, bladder neck mobility, GLU, postoperative analgesia, CCI, preoperative VTE score, Parity, and three types of surgery. After comparing six machine learning models, the GBDT model demonstrated the best predictive performance, achieving AUC values of 0.853 and 0.848 in the training set and test set, respectively, the model achieved stable prediction accuracy across multiple datasets, confirming its strong generalization ability. This finding is consistent with current research. Compared with single predictors, models combining patient baseline data and surgical factors have higher predictive value for the incidence of POUR (6).

Studies have shown that the POP-Q staging system based on objective assessment is an independent risk factor for predicting POUR (27). In this study, the Ba point value in the POP-Q system showed a significant correlation with the prediction of POUR risk. Patients often suffer from impaired urination due to anatomical abnormalities of the bladder neck (28), This suggests that Ba point can serve as an effective anatomical predictor for postoperative voiding dysfunction. Among the surgical types, consistent with previous studies, the risk of POUR was significantly increased when multiple prolapse repair procedures were performed (29–34). In particular, combined anterior and posterior repair surgery (35), carries a significantly higher risk of POUR than posterior colporrhaphy alone, which is related to the anatomical and physiological mechanisms that more extensive surgical procedures may cause pelvic floor nerve injury and changes in bladder neck position. Other surgical procedures such as sacrospinous ligament suspension were also included in the model, indicating the importance of individualized risk assessment based on preoperative anatomical evaluation and specific surgical combinations.

The CCI was confirmed to have predictive value in this study. As an effective tool for assessing patients’ comorbidity burden and long-term prognosis, the predictive performance of CCI has been validated in various surgical fields, including general surgery and cardiac surgery (36–38). In the field of pelvic floor surgery, previous studies have confirmed that comorbidity burden is a strong risk factor for postoperative complications. For example, one study showed that an elevated CCI score was closely associated with failure of the first postoperative voiding trial, increasing the risk of voiding dysfunction by 41%, and was therefore identified as an independent risk factor for voiding trial failure (39). Against this background, the present study focused on POUR as a specific complication in the context of pelvic floor reconstructive surgery and confirmed a positive correlation between CCI and POUR. This may be attributed to the fact that patients with multiple chronic diseases (such as diabetes and cardiovascular diseases) exhibit reduced tissue healing capacity and impaired neuromodulatory function, as well as generally compromised tolerance to surgical stress. In particular, diabetic patients may develop autonomic neuropathy, which directly impairs detrusor contractility and bladder sensation as a specific manifestation of impaired neuromodulatory function (40). Following pelvic floor surgery, they are more prone to developing bladder emptying dysfunction. Therefore, the systematic quantification of comorbidity burden incorporated into the preoperative assessment process is crucial for accurately identifying patients at high risk of POUR. Beyond preoperative risk assessment, long-term postoperative surveillance is equally important. For long-term management after POP reconstructive surgery, regular follow-up is essential to monitor for potential complications such as prolapse recurrence, mesh-related issues, and voiding dysfunction. In the context of other pelvic floor procedures, Alatawi et al. recommended the establishment of a comprehensive registry to facilitate systematic follow-up visits and collection of outcome data, thereby enhancing the evidence base regarding long-term efficacy and safety. We believe a similar approach could be applied to POP surgery to improve long-term patient outcomes (41).

Machine learning techniques have been widely applied in the field of postoperative complication prediction, and their advantages in handling high-dimensional data and complex clinical scenarios have been confirmed by several studies (42–44). In this study, GBDT achieved a higher AUC than logistic regression (ΔAUC = 0.0397). Although the AUC improvement was modest, it remained statistically significant after multiple comparison correction and stable across bootstrap resampling. GBDT also showed a lower Brier score and consistently higher net benefit across thresholds of 0.10–0.50. Thus, GBDT selection was supported by multiple performance dimensions rather than AUC ranking alone. Logistic regression remains a useful alternative when simplicity and transparency are prioritized. Given the single-center temporal validation, further prospective multicenter validation is required. In addition, this study introduced the SHAP method to conduct interpretability analysis of the model. By calculating the marginal contribution of each feature to the prediction results, SHAP quantifies the direction and magnitude of the influence of each variable in different individuals, and decomposes the overall prediction results of the model into visualizable feature attributions. In this study, SHAP analysis revealed that factors such as Ba point, CCI, and surgical procedure exerted significant impacts on POUR prediction, and their contribution directions were generally consistent with clinical experience. For instance, a greater degree of prolapse and a higher comorbidity burden corresponded to a higher risk of POUR. This consistency enhances the credibility of the model and provides an intuitive reference for clinicians to understand the basis of the model’s predictions. This study has the following advantages. First, this study compared the performance of various machine learning algorithms in predicting POUR after POP surgery, providing empirical evidence for model selection. Second, the sample size was relatively adequate (698 cases), with comprehensive collection of variables covering demographic characteristics, medical history, surgical procedures, pelvic examination findings, and laboratory indicators, which could reflect the patients’ clinical conditions in a relatively comprehensive manner. Third, the SHAP method was used to interpret the model, which not only improved model transparency but also provided a basis and direction for subsequent simplified modeling and screening of core variables. Fourth, in addition to using AUC to assess the discrimination of the model, calibration curves and decision curves were plotted for multi-dimensional evaluation, which more comprehensively reflects the clinical practical value of the model and aligns the results with the actual needs of clinical decision-making. Nevertheless, this study has several limitations. As a retrospective study, the completeness and accuracy of data recording may be limited. In addition, several well-established risk factors for POUR, including anesthetic agents, intraoperative fluid volume, operative time, analgesic protocol details, and duration of indwelling catheterization, were not available in our retrospective dataset, their absence may have introduced bias into our model estimates. Future prospective studies should incorporate these variables to improve model robustness. Furthermore, although temporal validation was performed, the lack of independent multicenter external validation limits generalizability and raises concern for potential overfitting. And all data were derived from a single tertiary center, which may not represent diverse patient populations with different baseline characteristics. Despite these limitations, this study provides valuable insights into the risk prediction of POUR. Future studies with multicenter prospective designs and larger sample sizes are warranted to further enhance the model’s generalizability and clinical applicability.

## 5 Conclusion

This study developed and temporally validated a GBDT machine learning model for predicting the risk of POUR following POP surgery. This model takes into account significant predictive parameters such as the CCI, surgical treatment type and severity of preoperative POP. Internal validation showed that it performed well in terms of discrimination, calibration, and clinical utility. The study’s findings confirm that the CCI is a good predictor of POUR, it indicates that incorporating the overall health status of patients into preoperative risk assessment is crucial. Other key predictive factors, such as the Ba value and specific surgical procedures, also provide clinicians with more targeted risk warnings and priorities for postoperative management. These findings support its selection as the final candidate model for further evaluation, while multicenter prospective external validation is still required before routine clinical application.

## Data Availability

The raw data supporting the conclusions of this article will be made available by the authors, without undue reservation.
