# Supplementary material for: Development and validation of a predictive model for postoperative urinary retention following pelvic organ prolapse surgery: a retrospective study
Source: Front Med (Lausanne). 2026 Jun 24;13:1851119. doi: 10.3389/fmed.2026.1851119 (PMC13341920; doi:10.3389/fmed.2026.1851119)
Supplement: Supplementary file 1 [file Data_Sheet_1.pdf]

Supplementary material

Supplementary Tables S1-S4

Table S1. Distribution of missing data for each candidate variable.

Variable	Missing Rate (%)
Preoperative VTE score	3.295
Bladder neck mobility	2.006
Postoperative analgesia	1.433
GLU (preoperative blood glucose)	0.573
AFB (age at first birth)	0.428
All other variables	0

Table S2. Sensitivity analysis of the selected GBDT model according to the use of SMOTENC oversampling in the training cohort.

Training strategy	Positive:negative ratio	AUC (95% CI)	Brier score (95% CI)	Sensitivity (95% CI)	Specificity (95% CI)
SMOTENC oversampling	1:2.00	0.848 (0.790-0.900)	0.143 (0.112-0.183)	0.571 (0.422-0.714)	0.861 (0.803-0.913)
NO oversampling	1:2.98	0.840 (0.779-0.890)	0.145 (0.112-0.182)	0.510 (0.362-0.660)	0.889 (0.837-0.934)

Table S3. Performance comparison of GBDT against other machine learning models in the temporal validation cohort.

Comparison	AUC of GBDT	AUC of Comparator	ΔAUC	95% CI	Raw P value	Holm-adjusted P value
GBDT vs XGBoost	0.8478	0.8253	0.0225	0.0098 to 0.0352	0.0005	0.0025
GBDT vs Logistic Regression	0.8478	0.8081	0.0397	0.0165 to 0.0629	0.0008	0.0032
GBDT vs LightGBM	0.8478	0.8367	0.0111	0.0035 to 0.0186	0.0040	0.0120
GBDT vs SVM	0.8478	0.8254	0.0224	0.0049 to 0.0399	0.0120	0.0240
GBDT vs Random Forest	0.8478	0.8410	0.0068	0.0003 to 0.0133	0.0410	0.0410

Table S4. Bootstrap comparison of GBDT versus Logistic Regression: AUC, Brier score, and net benefit.

Reference model	Comparison model	Metric	Difference definition	Interpretation	Observed difference	Bootstrap mean	Bootstrap SD	95% CI lower	95% CI upper	Bootstrap probability favoring reference	Reference favored in majority	CI excludes 0 in favor of reference	Bootstrap iterations
GBDT	Logistic Regression	AUC difference	GBDT - Logistic Regression	Higher favors reference	0.0397	0.0397	0.0101	0.0204	0.0607	1	TRUE	TRUE	1000
GBDT	Logistic Regression	Brier score difference	GBDT - Logistic Regression	Lower favors reference	-0.0057	-0.0057	0 .0013	-0.0083	-0.0030	1	TRUE	TRUE	1000
GBDT	Logistic Regression	Net benefit difference at threshold 0.10	GBDT - Logistic Regression	Higher favors reference	0.0086	0.0086	0.0021	0.0043	0.0124	1	TRUE	TRUE	1000
GBDT	Logistic Regression	Net benefit difference at threshold 0.20	GBDT - Logistic Regression	Higher favors reference	0.0013	0.0013	0.0004	0.0005	0.00212	1	TRUE	TRUE	1000
GBDT	Logistic Regression	Net benefit difference at threshold 0.30	GBDT - Logistic Regression	Higher favors reference	0.0215	0.0215	0.0070	0.0081	0.0357	1	TRUE	TRUE	1000
GBDT	Logistic Regression	Net benefit difference at threshold 0.40	GBDT - Logistic Regression	Higher favors reference	0.0242	0.0242	0.0073	0.0096	0.0384	1	TRUE	TRUE	1000
GBDT	Logistic Regression	Net benefit difference at threshold 0.50	GBDT - Logistic Regression	Higher favors reference	0.0052	0.0052	0.0016	0.0022	0.0084	1	TRUE	TRUE	1000

Supplementary Figure S1-S2

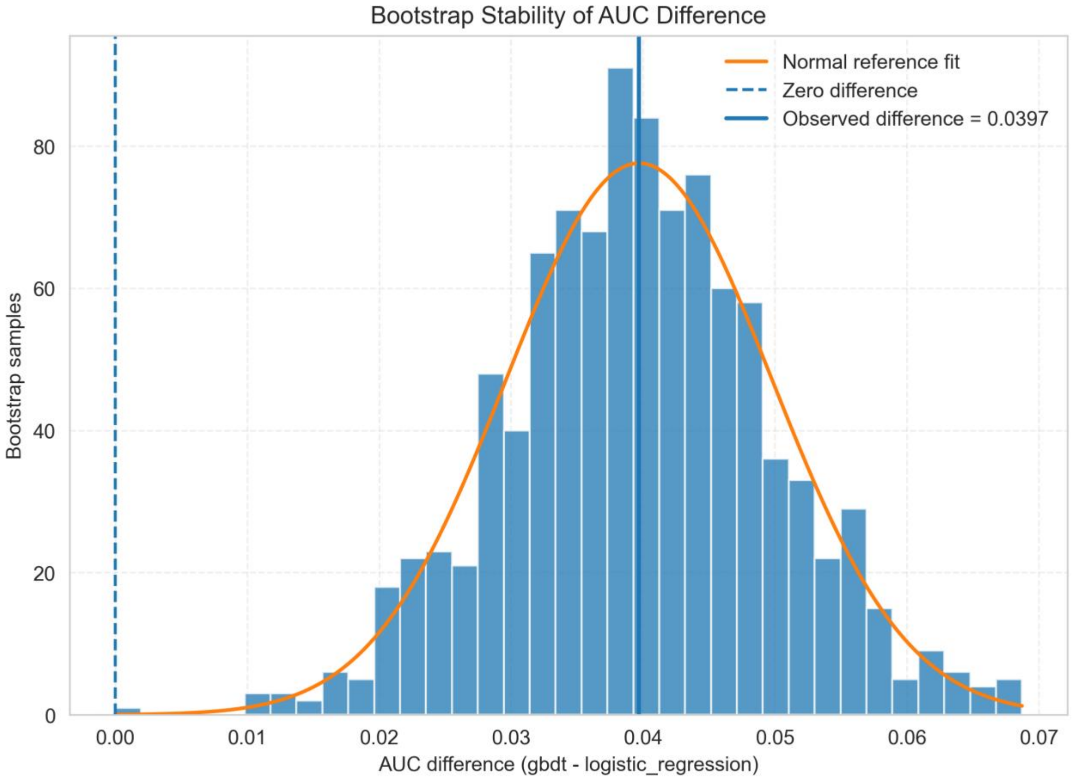

Figure S1：Bootstrap distribution of the AUC difference between GBDT and Logistic Regression.

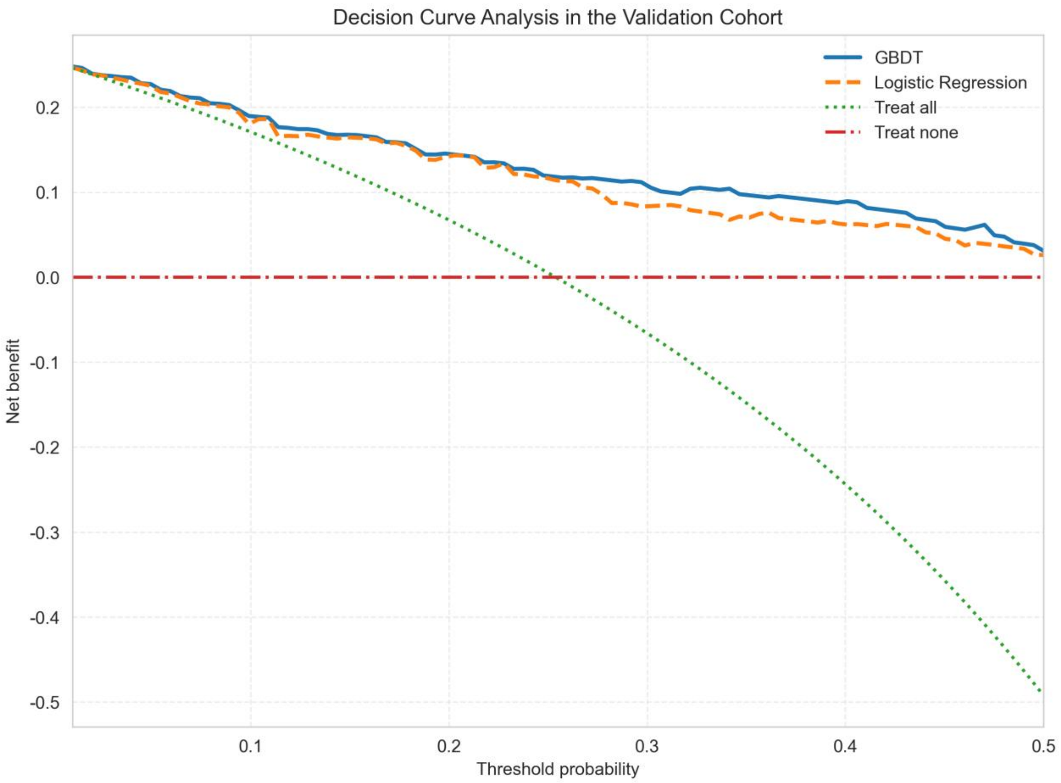

Figure S2： Direct decision curve analysis comparing GBDT with Logistic Regression.
